# Disruption of the GDP-mannose synthesis pathway in *Streptomyces coelicolor* results in antibiotic hyper-susceptible phenotypes

**DOI:** 10.1099/mic.0.000636

**Published:** 2018-03-01

**Authors:** Robert Howlett, Katri Anttonen, Nicholas Read, Margaret C. M. Smith

**Affiliations:** ^1^​ Department of Biology, University of York, York, UK; ^2^​ Institute of Medical Sciences, University of Aberdeen, Aberdeen, UK

**Keywords:** GDP-mannose pyrophosphorylase, phosphomannomutase, antibiotic resistance, phage resistance, polyprenol phosphate mannose

## Abstract

Actinomycete bacteria use polyprenol phosphate mannose as a lipid linked sugar donor for extra-cytoplasmic glycosyl transferases that transfer mannose to cell envelope polymers, including glycoproteins and glycolipids. We showed recently that strains of *Streptomyces coelicolor* with mutations in the gene *ppm1* encoding polyprenol phosphate mannose synthase were both resistant to phage φC31 and have greatly increased susceptibility to antibiotics that mostly act on cell wall biogenesis. Here we show that mutations in the genes encoding enzymes that act upstream of Ppm1 in the polyprenol phosphate mannose synthesis pathway can also confer phage resistance and antibiotic hyper-susceptibility. GDP-mannose is a substrate for Ppm1 and is synthesised by GDP-mannose pyrophosphorylase (GMP; ManC) which uses GTP and mannose-1-phosphate as substrates. Phosphomannomutase (PMM; ManB) converts mannose-6-phosphate to mannose-1-phosphate. *S. coelicolor* strains with knocked down GMP activity or with a mutation in *sco3028* encoding PMM acquire phenotypes that resemble those of the *ppm1*^-^ mutants i.e. φC31 resistant and susceptible to antibiotics. Differences in the phenotypes of the strains were observed, however. While the *ppm1*^-^ strains have a small colony phenotype, the *sco3028 *:: Tn*5062* mutants had an extremely small colony phenotype indicative of an even greater growth defect. Moreover we were unable to generate a strain in which GMP activity encoded by *sco3039* and *sco4238* is completely knocked out, indicating that GMP is also an important enzyme for growth. Possibly GDP-mannose is at a metabolic branch point that supplies alternative nucleotide sugar donors.

## Introduction

*Streptomyces* spp. are prolific producers of secondary metabolites, many with potent antibiotic activity. In nature *Streptomyces* spp. produce antibiotics either to inhibit competitors thus providing the producer with a growth advantage or as signalling molecules in microbial communities [1, 2]. Either way *Streptomyces* bacteria are constantly exposed to antibiotics produced by other soil microorganisms and consequently have evolved resistance mechanisms [3]. As such *Streptomyces* spp. are a model system to study how the mechanisms of antibiotic resistance evolve in an environmental organism.

**Fig. 1.**
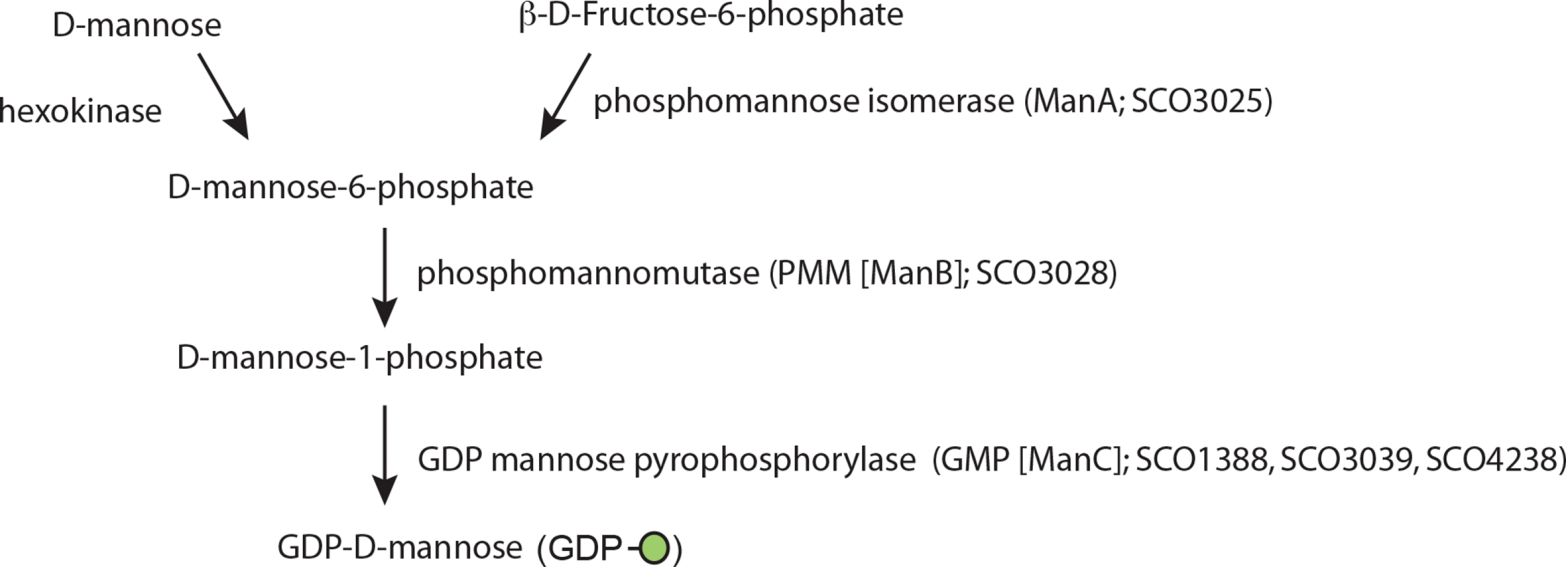
The GDP-mannose biosynthesis pathway in *Streptomyces coelicolor*.

We recently showed that strains of *S. coelicolor* lacking the ability to synthesise polyprenol phosphate mannose due to mutations in polyprenol phosphate mannose synthase (Ppm1) were hyper-sensitive to multiple antibiotics (Howlett *et al*., [4]). We used RNA-seq and Raman spectroscopy to demonstrate that the strains had undergone changes to the membrane phospholipids, with possible subsequent changes to membrane functions. Polyprenol phosphate mannose synthase, Ppm1, transfers mannose from GDP-mannose to polyprenol phosphate (Fig. 1). Previously we demonstrated that the synthesis of polyprenol phosphate mannose was entirely dependent on membrane associated Ppm1 [5].

Polyprenol phosphate mannose is the mannose donor for extracytoplasmic glycosyl transferases. One of these is a protein mannosyl transferase (Pmt), which glycosylates periplasmic and membrane proteins in *Streptomyces* [5, 6]. Pmt defective strains also show increased antibiotic susceptibility compared to the parent strain, but to fewer antibiotics and to a lower level than the *ppm1*^-^ mutants (Howlett *et al.* [4]). Loss of protein glycosylation is therefore likely to contribute in part to the antibiotic hyper-susceptible phenotype of the *ppm1*^-^ mutants. In addition both *ppm1*^-^ strains and the *pmt*^-^ strains are resistant to the phage φC31, most likely through loss of the receptor, although the exact nature of the phage receptor is still unknown [6, 7].

Polyprenol phosphate mannose is likely to be a mannose donor for other cell envelope macromolecules with one of these likely to be phosphoinositol mannosides (PIMs) [8, 9]. In other Actinobacteria including *Mycobacterium* and *Corynebacterium* spp. PIMs are precursors for the synthesis of lipoarabinomannan and lipomannan [10], but neither of these polymers has been reported in *Streptomyces*. Ppm1 is an essential enzyme in mycobacteria and a *ppm1*^-^ strain of *Corynebacterium* is growth retarded indicating the central role polyprenol phosphate mannose has in both organisms [11, 12]. The protein O-glycosylation pathway is present in most Actinobacteria and Pmt in Mtb has been shown to be important for virulence [13, 14]. In *Streptomyces coelicolor* other putative glycosyl transferases are also likely to use polyprenol phosphate mannose as a sugar donor and some of these are described in Howlett *et al.* [4].

The role of polyprenol phosphate mannose in antibiotic resistance and the pathway leading to its synthesis is addressed further in this paper (Fig. 1). d-mannose is either taken up from the medium and converted by hexokinase to d-mannose-6-phosphate or the latter can be produced from d-fructose-6-phosphate by phosphomannoisomerase (ManA). Phosphomannomutase (ManB:PMM) then converts d-mannose-6-phosphate to d-mannose-1-phosphate which is a substrate for GDP-mannose pyrophosphorylase (ManC:GMP). In *Corynebacterium glutamicum* deletion of the *manC* homologue (NCgl0710) conferred retarded growth and loss of nearly all mannoglycans from the envelope [15]. This phenotype resembles that of the *ppm1* mutant of *C. glutamicum* [11] and suggests that the ManB, ManC pathway is responsible for the synthesis of GDP-mannose. We hypothesised that strains containing blocks in the pathway leading to the synthesis of GDP-mannose ought to be phenotypically similar to the *ppm1*^-^ strains as they too will be deficient in polyprenol phosphate mannose. Here we analysed the roles of three putative *manC* genes in the *S. coelicolor* genome and a *manB* gene. We show that both a GMP depleted strain and a strain lacking PMM do indeed have phenotypes reminiscent of the *ppm1*^-^ mutants. The phenotype of the *S. coelicolor manB*^-^ strains constructed here varied from that reported previously for a *manB*^-^ strain [16, 17]. We conclude that GMP activity in *S. coelicolor* is provided by expression of two genes, *sco3039* and *sco4238*. Moreover both GMP and PMM activities are part of the same metabolic pathway leading to the synthesis of polyprenol phosphate mannose and ultimately to glycoprotein biosynthesis in *S. coelicolor*.

## Methods

### DNA manipulations

Chemically competent *E. coli* cells were prepared, stored and used in the transformation procedure as described previously [18]. Plasmid DNA extraction from *E. coli* was performed using a Spin Miniprep Kit following the protocol supplied by the manufacturer (QIAGEN). Cosmids were manipulated as described [19]. Restriction enzymes and T4 ligase were obtained from New England Biolabs (NEB) and used according to the manufacturer's instruction. Phusion High-Fidelity DNA Polymerase (NEB) was employed for PCR amplification. Primers used in the present study are listed (Table S1, available in the online version of this article). In-fusion HD cloning kit (Clontech) was used according to the protocol supplied by the manufacturer. DNA sequencing (Sanger) was outsourced to Source Bioscience.

### Plasmid, cosmid and strain constructions

A list of plasmids and cosmids used in this work is provided (Table 1). Plasmid pRH01 was produced by cloning of the PCR amplified product from primers RH11 and RH12 and J1929 genomic DNA as template, into EcoRV digested vector pAV11b [20–22]. Plasmid pRH12 was produced by cloning the PCR product from primers RH91 and RH92 and J1929 template into NdeI digested pIJ10257. Plasmids pRH11 and pRH14 were produced similar to pRH12 but using *E.coli* DH5α genomic DNA as template and primer pairs RH93/RH94, and RH140/RH141, respectively. Expression plasmids for *sco3039* (pRH06) and *sco4238* (pRH07) were produced through the ligation of XhoI and NdeI digested PCR products from primers pairs RH71/RH72, and RH73/RH74, respectively, and *S. coelicolor* J1929 template DNA, into XhoI and NdeI digested pET21a fusing both ORFs to an inframe C-terminal hexa-histidine tag. All constructs were confirmed as correct through Sanger sequencing performed by Source Bioscience.

**Table 1.** Bacteria, plasmids and cosmids

Plasmid name	Description	References
pAVIIb	Integrating vector with *tcp830* promoter and *tetRiS* cassette	[22]
pRH01	*sco4238* in pAV11b	This study
pDT16	*sco1423 (ppm1)* in pSET152	[7]
pDT10	*sco3154 (pmt)* in pSET152	[6]
pET21a	Overexpression vector containing HIS_6_-tag, T7 promotor	Merck Chemicals
pRH06	*sco3039* in pET21a	This study
pRH07	*sco4238* in pET21a	This study
pIJ10257	Integrating vector with constitutive promoter ermEp*	[40]
pRH11	*cpsG* in pIJ10257	This study
pRH12	*sco3028* in pIJ10257	This study
pRH14	*pgm* in pIJ10257	This study

Cosmid	Description	References
St1A8A.1.B09	SCO1388 :: Tn*5062* at nt 572	[28]
StD8A.2.D12	SCO4238 :: Tn*5062* at nt 69	[28]
StE34.1.G05	SCO3039 :: Tn*5062* at nt 155	[28]
StE34.1.B03	SCO3028 :: Tn*5062* at nt 590	[28]
StD8A.2.D12^spec^	StD8A.2.D12 with *sco4238 *:: Tn*5062*^spec^	This study
St1A8A.1.B09^hyg^	St1A8A.1.B09 with *sco1388 *:: Tn*5062*^hyg^	This study

*Streptomyces* strain	Genotype	References
M145	Prototroph	[23]
J1929	*pglY* mutant	[30]
DT3017	*ppm1*^E218V^ mutant	[7]
DT1020	*ppm1*^H116D^ mutant	[7]
DT1029	*ppm1*^S163L^ mutant	[7]
DT1025	*pmt,* frameshift from A121	[6]
DT2008	*pmt^-^*	[6]
SKA211	*sco3039 *:: Tn5062	This study
SKA311	*sco4238 *:: Tn5062	This study
RH501	*sco1388 *:: Tn5062	This study
RH25	*sco4238 *:: Tn5062^spec^	This study
RH221	*sco4238 *:: Tn5062^spec^, pRH01	This study
RH2213	*sco4238 *:: Tn5062^spec^ *sco3039 *:: Tn5062, pRH01	This study
RHB42	*sco3039 *:: Tn5062	This study
RHB4211	*sco3028 *: :Tn5062, pRH11	This study
RHB4212	*sco3028 *:: Tn5062, pRH12	This study
RHB4214	*sco3028 *:: Tn5062, pRH14	This study

*E.coli* strain	Genotype	References
ET12567(pUZ8002)	*dam- dcm- hsdS*, RP4 transfer genes	[41]
BL21 (DE3)	*lon, ompT, gal, hsdS*, λDE3	[18]
DH5α	F- Φ80*lac*ZΔM15 Δ(*lac*ZYA-*arg*F) U169 *rec*A1 *end*A1 *hsd*R17 (rk-, mk+) *pho*A *sup*E44 λ-*thi*-1 *gyr*A96 *rel*A1	[18]

The apramycin resistance markers within the Tn*5062* transposon of StD8A.2.D12 and St1A8A.1.B09 were replaced with spectinomycin and hygromycin markers, respectively, using the REDIRECT methodology [19]. Cosmids were introduced into *S. coelicolor* J1929 by conjugation and resistant exconjugants were selected according to the marker on Tn*5062* (apramycin, spectinomycin or hygromycin resistance). Those that had undergone a double cross over recombination event were identified initially as they lost the marker (kanamycin resistance) on the cosmid vector backbone. Presence of the interrupted allele and loss of the wild-type allele was confirmed by PCR and Southern blotting.

### Phage sensitivity assays

Plaque assays were performed as described [23]. Briefly Difco nutrient agar supplemented with 10 mM MgSO_4_ and 8 mM Ca (NO_3_)_2_ were inoculated with dilutions of φC31 *Δc25* (clear plaque) phage [24] and then overlayed with soft nutrient agar containing approximately 1×10^7^ spores of the desired test strain. The streak plate assay was performed using square 10 cm plates containing Difco nutrient agar [10 mM MgSO_4_ and 8 mM Ca (NO_3_)_2_]. One half of the plate was inoculated with 100 µl of φC31 *Δc25* (approx 1×10^8^ p.f.u. ml^−1^) and a single streak of the test spore preparation was inoculated across the plate beginning on the phage-free region. Plates were incubated at 30 °C.

### Protein expression

An overnight culture of *E. coli* BL21DE3 (pRH07) in LB containing ampicillin was grown at 37 °C and used to inoculate 2YT, which was grown to OD 0.6. IPTG (0.15 mM) was then added to induce expression and the culture was further incubated (22 °C for 22 h). The bacteria were harvested by centrifugation and resuspended in binding buffer (30 ml; 20 mM Tris HCl pH 7.4, 0.5M NaCl, 30 mM imidazole) and sonicated. The cell lysate was cleared by centrifugation (4 °C, 5 min, 10 000 ***g***) to remove unlysed cells and debris and then the supernatant was loaded onto a HiTrap Ni^2+^ affinity column (AKTA Purifier). After washing with 2 column volumes of binding buffer the bound protein was eluted with a gradient of increasing imidazole concentration using the elution buffer (20 mM Tris HCl pH 7.4, 0.5M NaCl, 500 mM imidazole). Pooled fractions were then loaded onto a desalting column to remove imidazole and eluted in 20 mM Tris HCl pH 7.4, 0.5M NaCl. The protein was concentrated using Vivaspin (GE healthcare) spin columns to approximately 10 mg ml^−1^. Glycerol was added to a final concentration of 50 % and aliquots were stored at −80 °C. Protein concentration was assayed using the BioRad protein assay solution and is based on the Bradford assay [25].

### GDP mannose pyrophosphorylase assays

Activity was measured by monitoring the release of pyrophosphate using the EnzChek pyrophosphate assay kit (Thermofisher). Briefly, the kit includes a pyrophosphatase that catalyses the conversion of the pyrophosphate released from the GMP activity to two equivalents of phosphate, which is then used as a substrate in a reaction with 2-amino-6-mercapto-7-methylpurine ribonucleoside (MESG) and purine nucleoside phosphorylase (PNP) to release ribose 1-phosphate and 2-amino-6-mercapto-7-methyl-purine. The latter compound was detected spectrophotometrically by absorbance at 360 nm. Assays were performed according to the manufacturer's instructions except that they were scaled down to enable use of a 96 well plate reader (200 µl assay volume per well). GMP activity rates were obtained using different nucleotides (1 mM ATP, GTP, CTP or dTTP) and sugars, (1 mM mannose-1-phosphate or mannose-6-phosphate) as substrates. Initial rates were calculated and plotted against substrate concentration using SIGMAplot.

## Results

### Identification of putative GDP-mannose pyrophosphorylases

**Fig. 2.**
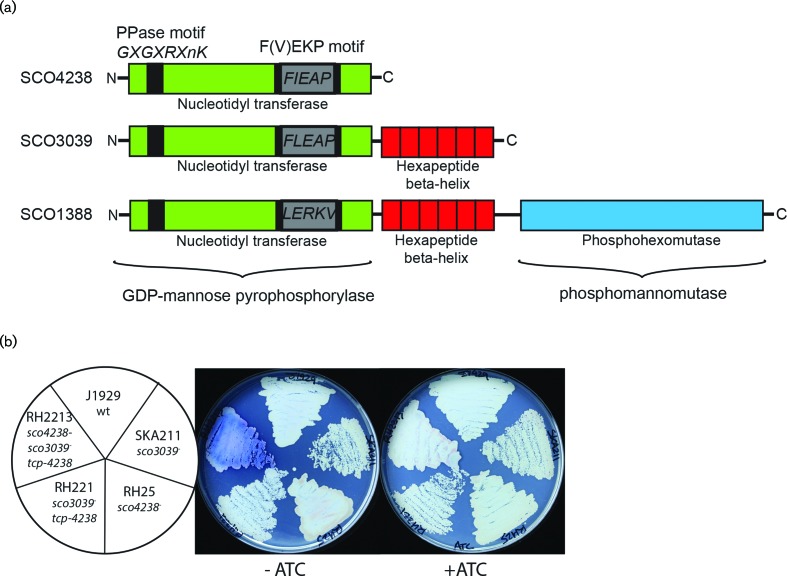
GDP-mannose pyrophosphorylases in *Streptomyces coelicolor* (a). Domain structures for *S. coelicolor* genes with putative GDP-mannose phosphorylase activity. (b). Pigment overproduction in *sco4238, sco3039* double mutants. Strain RH2213 (*sco4239 :: Tn5062spec*, *sco3039 :: Tn5062,* pRH01 encoding inducible *sco4238)* overproduced blue pigment on supplemented minimal medium solid (SMMS) agar in the absence of anhydrotetracycline (ATC) but not in the presence of 0.5 µg ml^−1^ ATC.

GDP-mannose, a substrate for Ppm1, is synthesised by GDP-mannose pyrophosphorylase (GMP) encoded by *manC* (Fig. 1). blast searches of the *Streptomyces coelicolor* genome with the characterised *Corynebacterium glutamicum* GMP/ManC (encoded by NCgl0710) and *Mycobacterium tuberculosis* GMP/ManC (encoded by Rv3264c) identified SCO1388, SCO3039 and SCO4238 as putative GMP candidates (Fig. 2) [15]. The nucleotidyl transferase domains of all three *Streptomyces* GMP/ManC candidates contain the GXGXRXnK signature motif of phosphorylases, and variations on the F(V)EKP motif characteristic of the GMP active site (Fig. 2) [26, 27]. SCO3039 and SCO1388 have protein domains in addition to the nucleotidyl transferase domain; SCO1388 in particular appears to be a bifunctional enzyme with both GMP and phophomannomutase (ManB) activity (Fig. 2).

### SCO3039 and SCO4238 have overlapping functions

The *S. coelicolor* genes *sco1388*, *sco3039* and *sco4238* were disrupted by allelic exchange with cosmids containing Tn*5062* in the gene of interest to produce single insertion mutants RH501 (*sco1388 *:: Tn*5062*), SKA211 *(sco3039 *:: Tn*5062*) and SKA311 (*sco4238 *:: Tn*5062*). The cosmids were obtained from the transposon insertion cosmid library [28] (Table 1). Validated mutants were tested for φC31 resistance as a potential indicator for a loss of GMP/ManC activity due to a lack of protein *O*-glycosylation [6, 7], but all of the mutants were still sensitive to phage infection. A mild increase in blue pigment production was seen in the mutant strains SKA311 (*sco4238 *:: Tn*5062*) and SKA211 (*sco3039 *:: Tn*5062*) when grown on supplemented minimal media (SMM) (not shown).

Double mutants were created to assess whether there is redundancy in gene function between *sco3039, sco4238* and *sco1388*. The cosmid StD8A.2.D12^spec^ (*sco4238*:Tn*5062^spec^*) was introduced into J1929 by conjugation to create the spectinomycin resistant *sco4238* insertion mutant, RH25. The cosmid StE34.1.G05 (*sco3039 *:: Tn*5062*) was then introduced into RH25 by conjugation, selecting for apramycin resistance. Only eight exconjugants from several hundred that were screened had the spectinomycin-resistant, apramycin-resistant, kanamycin-sensitive phenotype indicative of a *sco4238 :: *Tn*5062^spec^*, *sco3039 :: *Tn*5062* double mutant. However subsequent analysis by polymerase chain reaction (PCR) to amplify the genomic region containing *sco3039* showed that this gene was uninterrupted in all eight candidate double mutant strains and mutations must have occurred elsewhere to confer resistance to apramycin. Thus we were unable to create a simple double mutant containing Tn*5062* insertions in both *sco3039* and *sco4238,* suggesting that these genes share an important function for growth. Multiple *sco4238 :: Tn5062^spec^, sco1388 :: Tn5062* and *sco3039 :: Tn5062, sco1388 :: Tn5062^hyg^* double mutant strains were produced and confirmed through kanamycin sensitivity. The phenotypes of these strains were no different from the individual *sco4238 :: Tn5062* and *sco3039 :: Tn5062* mutants, SKA311 and SKA211, respectively. The product of *sco1388* therefore probably contributes little to the total GMP activity in *S. coelicolor.*

We were able to create a strain containing both *sco4238 :: Tn5062^spec^* and *sco3039 :: Tn5062* insertions in the presence of a conditionally expressed *sco4238*. Plasmid pRH01, encoding *sco4238* under the control of the anhydrotetracycline (ATC) inducible promoter, *tcp830* [21], was introduced into RH25 to create strain RH221 (*sco4238 *:: Tn*5062^spec^*, *tcp830-sco4238, hyg*). Conjugation of StE34.1.G05 (*sco3039 *:: Tn*5062*) into RH221 in the presence of ATC resulted in multiple spec^R^, apra^R^, hyg^R^, kan^S^ exconjugants (RH2213) that were subsequently confirmed as *sco4238 :: *Tn*5062^spec^*, *sco3039 :: *Tn*5062* double mutants through PCR. Surprisingly RH2213 could grow in the absence of ATC, an observation that was at odds with our inability to isolate the transposon double mutants in the absence of pRH01. Colony sizes of the RH2213 strains in the absence of ATC were indistinguishable from the wild-type parent strain, J1929, but a significant increase in blue pigments were observed compared to the single mutants RH25 (*sco4238 *:: Tn*5062^spec^*) and SKA211 *(sco3039 *:: Tn*5062*)(Fig. 2b). The *tcp830* promoter has been shown by others to be incompletely turned off in the absence of ATC and we propose that this is also the case in our experiments [20]. It seems likely that RH2213 grown in the absence of ATC has a depleted level of GMP compared to the parent strain and compared to RH2213 grown in the presence of ATC.

### Strains depleted in the putative GMPs SCO3039 and SCO4238 are hyper-susceptible to antibiotics and partially resistant to φC31

**Fig. 3.**
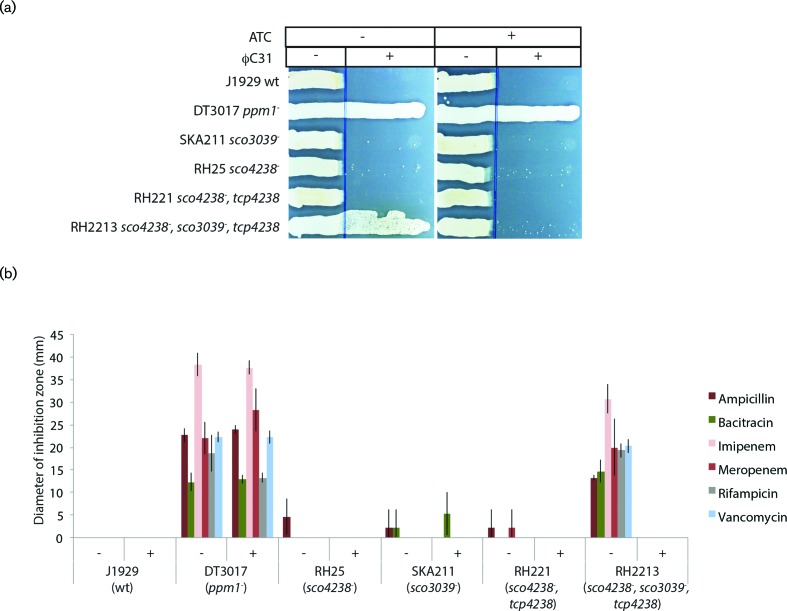
*Streptomyces coelicolor* strains depleted in GDP mannose pyrophosphorylase activity are partially resistant to φC31 and are hyper-susceptible to some antibiotics (a). Spores of the indicated *S. coelicolor* strains were streaked from an area free from φC31 to an area inoculated with 1×10^7^ p.f.u. φC31 on Difco nutrient agar plates with or without the supplementation of 0.5 µg ml^−1^ anhydrotetracycline (ATC). RH2213 (*sco4239 :: Tn5062^spec^*, *sco3039 :: Tn5062,* pRH01 encoding inducible *sco4238)* showed conditional phage resistance growing only in the absence of ATC. For comparison the phage resistant phenotype of the *ppm1^-^* mutant (DT3017), the parent strain (J1929) and the strains with single mutations in the *manC* candidate genes, *sco3039* and *sco4238*, (SKA211 and RH25, respectively). (b). RH2213 showed increased susceptibility to antibiotics in the absence of ATC but not in the presence of ATC. This phenotype is comparable to the antibiotic hyper-susceptible phenotype of the *ppm1^-^* mutant (DT3017). Results show the mean diameter of the disc diffusion inhibition zones from at least 3 replicates. Antibiotics were all used at 4 µg/disc with the exception of ampicillin that was used at 40 µg/disc.

Ppm1 uses GDP-mannose as a substrate and we therefore hypothesised that inability to synthesise GDP-mannose, for example through GMP depletion, should result in a similar phenotype to those strains deficient in Ppm1. RH2213 isolates (*sco4239 :: Tn5062^spec^*, *sco3039 :: Tn5062,* pRH01 encoding inducible *sco4238)* were still able to support φC31 plaque formation but displayed resistance to φC31 on a streak assay in the absence of ATC (Fig. 3a).

We then tested the putative GMP deficient strains for their susceptibilities to antibiotics, notably those to which the *ppm1* and *pmt* mutants were particularly sensitive. *S. coelicolor* strains SKA211 and RH25 containing Tn*5062* insertions in *sco3039* and *sco4238*, respectively, had the same antibiotic resistances as the parent strain J1929. However RH2213, with depleted levels of GMP in the absence of ATC, was highly susceptible to antibiotics, strongly resembling the phenotypes of the *ppm1* mutants (Fig. 3b). The phenotypes of the GMP depleted mutants indicate that *sco3039* and *sco4238* provide the majority of the GMP activity in *S. coelicolor*.

### *sco4238* encodes a highly specific GDP-mannose pyrophosphorylase activity

**Fig. 4.**
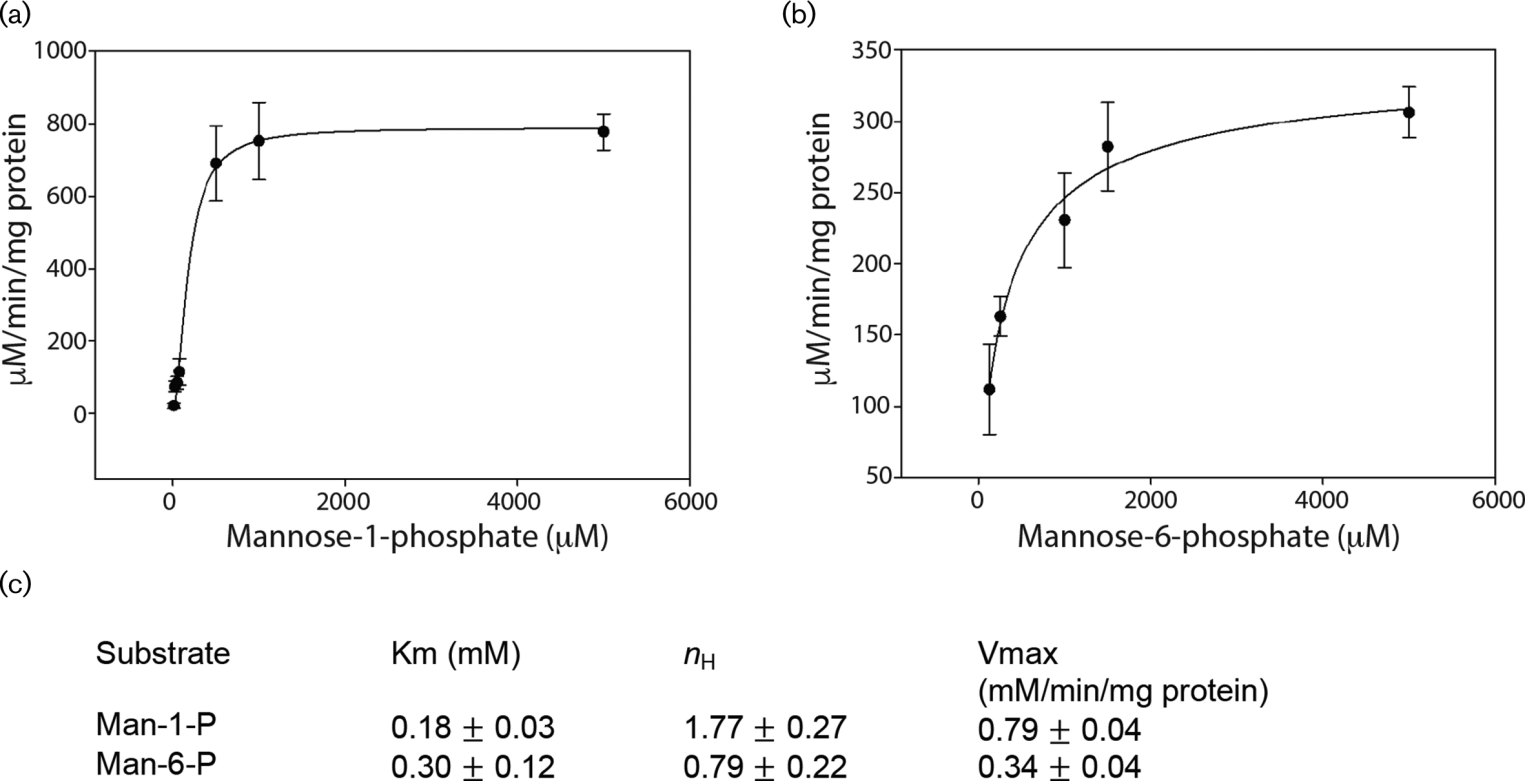
Kinetics of GDP-mannose pyrophosphorylase activity of SCO4238. Dependence of initial velocity of SCO4238 GMP activity with increasing concentration of mannose-1-phosphate (a) and mannose-6-phosphate (b) Kinetic parameters Km, *n*_H_ and Vmax for the two sugar phosphate substrates (c).

To confirm the phenotypes mentioned above were due to a depletion of GMP activity in RH2213, *sco4238* and *sco3039* were overexpressed in *E. coli* in order to assay GMP activity on purified proteins. Overproduced SCO4238 showed high GMP activity (Fig. 4). The enzyme was highly specific for GTP and d-mannose-1-phosphate substrates, with no or very low rates achieved with CTP, ATP and dTTP (not shown). Approximately 50 % activity was observed with d-mannose-6-phosphate and GTP, with the Hill coefficient showing a loss of the cooperativity seen with d-mannose-1-phosphate. In *Mycobacterium tuberculosis* the essential enzyme, RmlA, catalyses the synthesis of dTDP-glucose, an intermediate in dTDP-rhamnose biosynthesis required for the integrity of the cell wall [29]. Given the apparent essentiality of GMP in *S. coelicolor* we tested whether SCO4238 had activity on glucose-1-phosphate in combination with any nucleotide, including dTTP but no activity was detected. Attempts to obtain soluble, active SCO3039 from several overexpression constructs in *E. coli* failed (not shown).

### Strains with a mutation in the *manB* gene, *sco3028* are also phenotypically similar to the *ppm1* mutants

Previous work has shown that SCO3028 is a dual functioning enzyme capable of phosphomannomutase (PMM, mannose-6-phosphate to mannose-1-phosphate) and phosphoglucomutase (PGM, glucose-6-phosphate to glucose-1-phosphate) activity [17]. The authors constructed a *manB* deletion mutant, *ΔmanB,* which had increased actinorhodin production and had lost chloramphenicol resistance but displayed apparently similar growth to the parent strain, M145 [16, 17]. Both phenotypes of the *ΔmanB* strain were complemented when the wild-type *E. coli manB* was introduced whilst the *S. coelicolor pgm* gene (*sco7443*) failed to complement. Thus PMM activity was shown to be solely responsible for an increase in chloramphenicol sensitivity and actinorhodin production in *S. coelicolor* M145. If SCO3028 is the sole PMM enzyme in *S. coelicolor* we would expect a similar phenotype in the *sco3028* mutant as we see for the GMP depleted strains. However, Yang *et al.* did not detect increased susceptibility of their *ΔmanB* strain to vancomycin, bacitracin or ampicillin [17].

**Fig. 5.**
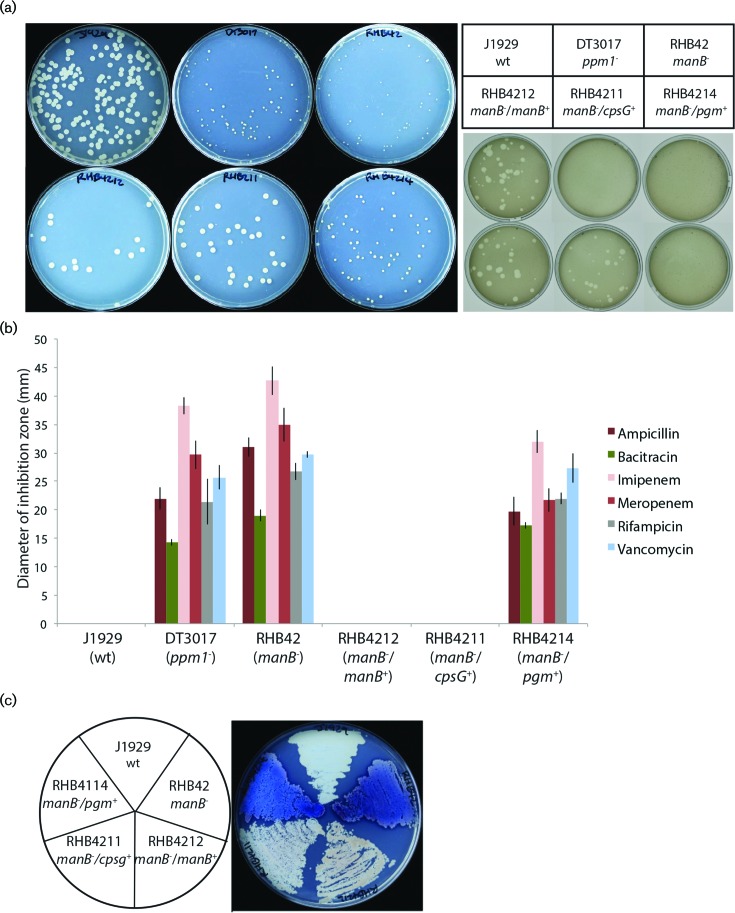
Phenotypes of the *manB*^-^ (*sco3028 *:: Tn*5062*) mutants. (a). Extreme *s*mall colony phenotype (left) and resistance to phage φC31 (right) in the RHB42 (*sco3028 *:: Tn*5062*) mutant strains. The poor growth of RHB42 was complemented with an additional copy of *sco3028* (RHB4212) or the *E. coli* gene *cpsG* (*manB* homologue; RHB4211) but not *pgm* from *E. coli* (encoding phosphglucomutase; RHB4214). The φC31 resistance in RHB42 reverted to phage sensitivity in the complemented strains RHB4211 and RHB4212. (b). RHB42 has increased susceptibility to antibiotics resembling the phenotype of the *ppm1*^-^ mutant (DT3017). Antibiotic susceptibility could be complemented with an additional copy of *sco3028* and *E. coli cpsG* but not *pgm* from *E. coli*. (c). Increased pigment production in RH42 compared to the parental strain, J1929. This phenotype was complemented by additional copy of *sco3028* and *E. coli cpsG* but not *pgm* from *E. coli*.

In order to assess the phenotype of an *sco3028* mutant in our φC31 sensitive strain *S. coelicolor* J1929, a *pglY*^-^ derivative of M145 [30], the cosmid StE34.1.B03 (*sco3028 *:: Tn*5062*) was introduced into J1929 by conjugation. Exconjugants that had undergone a double crossover (RHB42 strains, validated by PCR) were isolated at low frequency and had an extreme small (XS) colony phenotype, even smaller than the colony size seen in the *ppm1* mutant DT3017 (Fig. 5). The XS colony phenotype in RHB42 could be fully restored to wild-type through complementation with *S. coelicolor sco3028 (manB),* and *Escherichia coli manB* (*cpsG*) as observed in strains RHB4212 and RHB4211, respectively. RHB42 containing *Escherichia coli pgm,* encoding phosphoglucomutase, was capable of partially restoring colony size (RHB4214), suggesting it is the loss of both PMM and PGM activity that had resulted in the XS colony phenotype in RHB42.

RHB42 was highly resistant to φC31 infection in a plaque assay, resembling phage resistance in the *pmt* and *ppm1* mutants (Fig. 5a). RHB42 was also highly susceptible to a number of cell wall acting antibiotics, as well as the RNA polymerase targeting antibiotic, rifampicin (Fig. 5b). Phage sensitivity and antibiotic resistance were restored to wild-type in RHB4212 (*sco3028^+^*) and RHB4211 (*cpsG^+^*) but not in RHB4214 (*pgm^+^*). No difference in chloramphenicol resistance between RHB42 and J1929 was observed. These phenotypes are consistent with SCO3028 being the primary PMM in *S. coelicolor* and in the same metabolic pathway that synthesises polyprenol phosphate mannose.

An increase in pigment production was recorded in RHB42, similar to that seen previously following *sco3028* deletion [17] and that seen in the GMP depleted strain, RH2213 (Fig. 5c). The production of blue pigment was reduced to wild-type level in RHB4212 (*sco3028^+^*) and RHB4211 (*cpsG^+^*) but not RHB4214 (*pgm^+^*) (Fig. 5). To further validate our observations (as they differ from those of Yang *et al.* [16, 17]), we created two more sets of *sco3028 :: *Tn*5062* mutants: First we used a different Tn*5062* insertion in J1929 using cosmid, STE34.2.D03, generating strain JD182 and second we generated derivatives of M145 containing the Tn*5062* insertions from both STE34.1.B03 and STE34.2.D03 to generate strains MD202 and MB92, respectively. All three strains had an identical phenotype to RHB42 (Figs S1 and S2).

## Discussion

Mannose is a component of cell envelope polymers including mannolipids, phosphoinositol mannosides (PIMs) and glycoproteins in many bacteria [10, 31–33]. Extracytoplasmic glycosyl transferases use polyprenol phosphate mannose as the lipid linked sugar donor in the biosynthesis of mannose containing polymers [5, 34]. The synthesis of polyprenol phosphate mannose by Ppm1 is therefore an important activity and *ppm1* mutants are considerably less fit than the parent strains [11, 12]. In the case of *S. coelicolor, ppm1*^-^ mutants have a small colony growth phenotype and are hyper-susceptible to multiple antibiotics, most of which inhibit cell wall biogenesis suggesting that these mutants are pleiotropically deficient in membrane and/or periplasmic function (Howlett *et al.*, [4]). Mutants lacking Ppm1 or Pmt are also resistant to phage infection and we have proposed that φC31 uses a glycoprotein(s) as its receptor [6, 7]. We show here that depletion of enzymes in the mannose metabolism pathway prior to Ppm1 display a phenotype that resembles that of the *ppm1*^-^ mutants. We conclude that synthesis of polyprenol phosphate mannose and its subsequent role as a mannose donor in the periplasm is required for a wild-type antibiotic resistant phenotype.

Although the overall phenotypes of the GDP-mannose pyrophosphorylase (GMP) deficient and the *manB* mutant strains resembled the *ppm1*^-^ strain there were some minor differences. We had difficulty in generating a GMP deficient strain. *S. coelicolor* has three candidate genes that could express GMP activity and we could only obtain a double *sco3039^-^*, *sco4238^-^* mutant if *sco4238* was expressed conditionally using the anhydrotetracycline-inducible *tcp830* promoter. While this is not absolute proof that the GMP activity is essential in *S. coelicolor*, it would seem that some low level of activity, possibly that provided by the leakiness of the repressed *tcp830* promoter reading into an integrated copy of *sco4238*, is required for the simultaneous interruption of both *sco3039* and *sco4238* by Tn*5062*. Similarly the insertion in *sco3028* (*manB)* was obtained at very low frequency and the colonies were extremely small, indicative of a requirement for both phosphomannomutase (PMM) and phosphoglucomutase (PGM) activities encoded by this gene. If GMP activity is essential then we would also expect PMM activity to be essential, but there may be sufficient PMM activity from other closely related enzymes (such as other PGM paralogues; *sco7443* or *sco4916* a possible alternative phosphomannomutase) to allow growth. The more severe phenotypes of the GMP depletion mutant and the *manB* mutant also suggest that GDP-mannose could be located at a metabolic branch point i.e. GDP-mannose is required for polyprenol phosphate mannose synthesis but also perhaps for modification into other nucleotide sugars. For example, the *S. coelicolor* genome encodes a GDP-mannose dehydrogenase (SCO0382) that is predicted to make GDP-mannuronate, one of the building blocks in the synthesis of alginates in *Pseudomonas. Sco0382* lies within an operon *sco381* to *sco386* that has features of an extracellular polysaccharide biosynthesis gene cluster including a polyprenol dependent glycosyl transferase and various other membrane proteins.

Enzyme assays with purified SCO4238 showed it to be a monofunctional GMP (ManC) with a slim substrate tolerance similar to Rv3264 (previously miss-annotated as *rmlA*) of *Mycobacterium* [29, 35] and in contrast to the more promiscuous ManC enzymes of *E. coli* and *P. furiosis* [36, 37]. In *Mycobacterium* and in *Corynebacterium* the ManC enzymes (Rv3264 and NCgl0710, respectively) provide essential supplies of GDP-mannose for phosphatidyl inositol mannoside (PIM) biosynthesis and lipoglycans [15].

The phenotype described here for RHB42 (*sco3028*:Tn*5062*, *manB*^-^) has differences and similarities to a *ΔmanB* strain of *S. coelicolor* M145 that was described previously [16, 17]. A notable difference is the sensitivity to antibiotics of RHB42 as Yang *et al.* did not detect an increase in susceptibility of their *ΔmanB* strain to vancomycin, bacitracin or ampicillin [17]. Whilst we cannot explain these differences in phenotypes, both RHB42 and the *ΔmanB* of Yang *et al.* have increased pigment production. Pigment production is also greatly increased in the ManC deficient strains. The increase in pigment production could be indicative of the activation of several stress pathways or, as discussed by Yang *et al.*, could be due to the increase in carbon flux through glycolysis as the pathway to GDP-mannose is blocked. The *ppm1*^-^ strain DT3017 has a mild pigment overproduction phenotype (data not shown). Neither Yang *et al.*, or Rajesh *et al.* could test phage sensitivity in their *ΔmanB* strain as they used a Pgl^+^ strain of *S. coelicolor,* which confers φC31 resistance.

Mannose is used in other *Streptomyces spp* in the biosynthesis of antibiotics e.g. mannopeptimycins and amphotericin [38, 39]. ManB and ManC activities are required in *S. nodosus* for the glycosylation of amphotericin [39]. The identification of the *manC* genes and the construction of the *manC* deficient strain could be useful in heterologous expression and combinatorial biosynthesis of several antibiotic pathways in *S. coelicolor*.

## Supplementary Data

3557 Supplementary File 1
