# Supplementary material for: Disruption of the GDP-mannose synthesis pathway in Streptomyces coelicolor results in antibiotic hyper-susceptible phenotypes
Source: Microbiology (Reading). 2018 Mar 1;164(4):614–24. doi: 10.1099/mic.0.000636 (PMC5982138; doi:10.1099/mic.0.000636)
Supplement: Supplementary File 1 [file mic-164-614-s001.pdf]

M145<br>wt	MD202<br>*sco3028*$^{-}$	MD202<br>*sco3028*$^{-}$, *cpsG*$^{+}$	MD202<br>*sco3028*$^{-}$,*sco3028*$^{+}$	MD202<br>*sco3028*$^{-}$, *pgm*$^{+}$
	MB92<br>*sco3028*$^{-}$	MB92<br>*sco3028*$^{-}$, *cpsG*$^{+}$	MB92<br>*sco3028*$^{-}$,*sco3028*$^{+}$	MB92<br>*sco3028*$^{-}$,*pgm*$^{+}$
	JD182<br>*sco3028*$^{-}$	JD182<br>*sco3028*$^{-}$, *cpsG*$^{+}$	JD182<br>*sco3028*$^{-}$,*sco3028*$^{+}$	JD182<br>*sco3028*$^{-}$,*pgm*$^{+}$

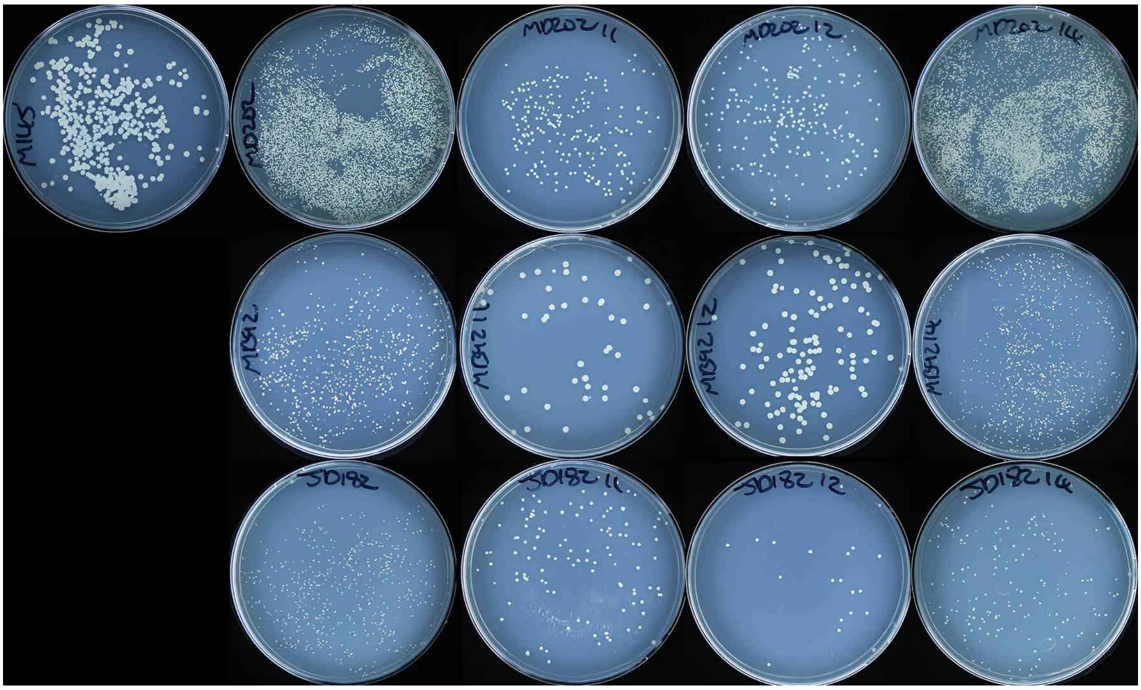

Figure S1

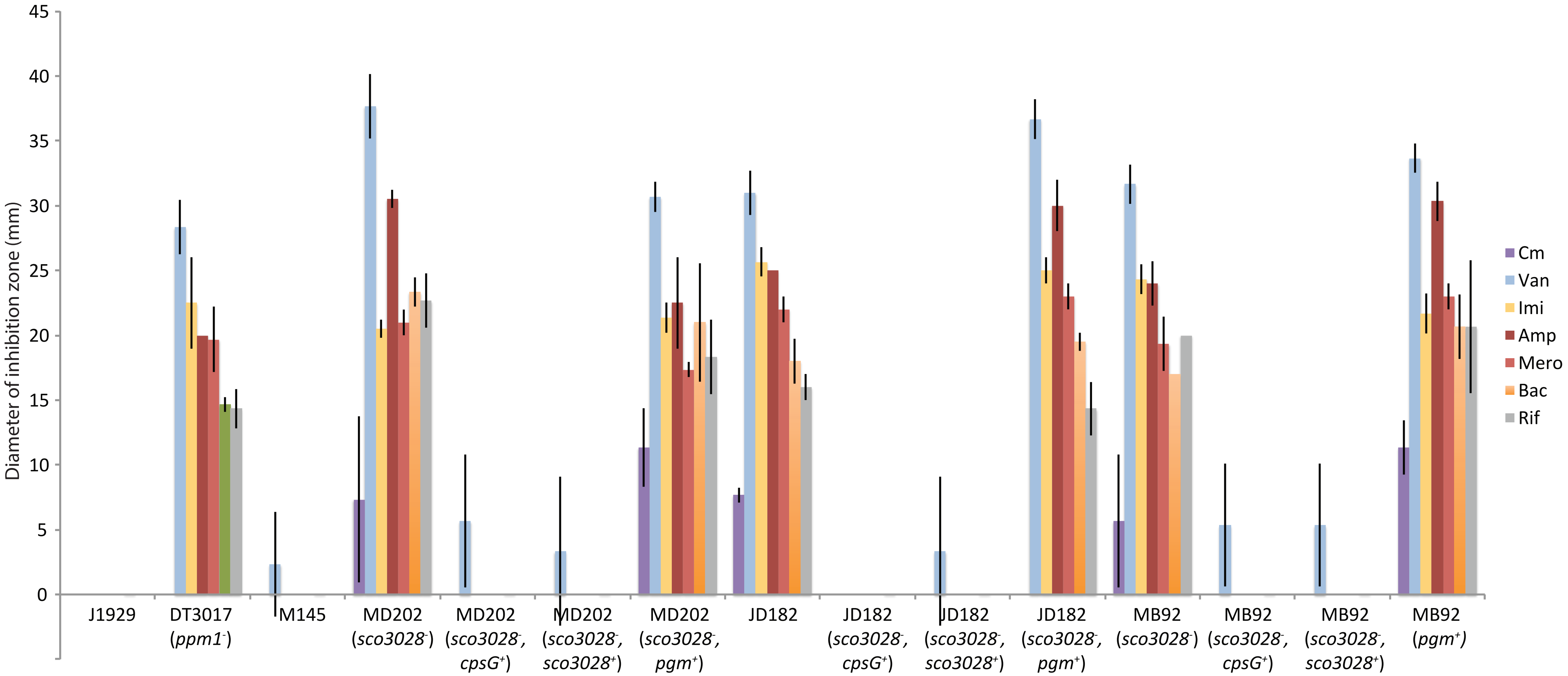
Diameter of inhibition zone (mm)
45
40
35
30
25
20
15
10
5
0
J1929
DT3017 (ppm1-)
M145
MD202 (sco3028-)
MD202 (sco3028-, cpsG+)
MD202 (sco3028-, sco3028+)
MD202 (sco3028-, pgm+)
JD182
JD182 (sco3028-, cpsG+)
JD182 (sco3028-, sco3028+)
JD182 (sco3028-, pgm+)
MB92 (sco3028-)
MB92 (sco3028-, cpsG+)
MB92 (sco3028-, sco3028+)
MB92 (pgm+)
Cm
Van
Imi
Amp
Mero
Bac
Rif

Figure S2

Supplementary Figure Legends. Howlett *et al.*

Figure S1. Extreme small colony phenotype of various *manB*⁻ strains of *S. coelicolor*. MD202 is a derivative of *S. coelicolor* M145 containing *sco3028::Tn5062* at nt 590 derived from cosmid StE34.1.B03 (1, 2). MB92 and JD182 are derivatives of *S. coelicolor* M145 and J1929, respectively, containing *sco3028::Tn5062* at nt 576 derived from cosmid StE34.2.D03 (1, 2). In each case a transposon insertion in *sco3028*, (encoding ManB) confers an extreme small colony phenotype. The phenotype can be fully complemented by introduction of a wild type copy of *sco3028* (*sco3028*$^+$ in pRH12), *E. coli cpsG* (encoding ManB in pRH11) but not by *E. coli pgm* (encoding phosphoglucomutase, pRH14).

Figure S2. Antibiotic hyper-susceptible phenotype of various *manB*$^-$ strains *of S. coelicolor*. Strains are as described in the legend to Figure S2. For comparison the antibiotic susceptibility profiles of the parent strains J1929 and M145 and that of the *ppm1*$^-$ strain DT3017 are also shown.

References

1. Bishop A, Fielding S, Dyson P, Herron P. Systematic insertional mutagenesis of a streptomycete genome: a link between osmoadaptation and antibiotic production. Genome Res. 2004;14(5):893-900.
2. Fernandez-Martinez LT, Del Sol R, Evans MC, Fielding S, Herron PR, Chandra G, et al. A transposon insertion single-gene knockout library and new ordered cosmid library for the model organism Streptomyces coelicolor A3(2). Antonie Van Leeuwenhoek. 2011;99(3):515-22.

Table S1. Oligonucleotides for Howlett, Read *et al*,

Primer	Sequence	Use
RH11	TCTAGAGACGTCGATATCATGACCGATCCGAACGCC GCGTCC	*sco4238* F Infusion; pRH01
RH12	CATGCATGATCAGATATCTCAGCGGCCGGACAGGG CCG	*sco4238* R Infusion; pRH01
RH71	GACGCCCATATGACAGAAGCGATCCTCCTG	*sco3039* (NdeI); pRH06
RH72	CGTTACAAGCTTCGTGTCCGGTGAGAAGCG	*sco3039* (HindIII); pRH06
RH73	GGCGCCCATATGACCGATCCGAACGCCGCGTC	*sco4238* (NdeI); pRH07
RH74	GTACCTCGAGGCGGCCGGACAGGGCCG	*sco4238* (XhoI); pRH07
RH91	ACAGGAGGCCCCATATGGCTGCTGATCTGTCGCAG	*sco3028* F Infusion; pRH12
RH92	ACTCGAGATCTCATATGTCACGCCCGGATGATCGCC	*sco3028* R Infusion; pRH12
RH93	ACAGGAGGCCCCATATGAAAAAATTAACCTGCTTT	*cpsG(manB)* F Infusion; pRH11
RH94	ACTCGAGATCTCATATGTTACTCGTTCAGCAACG	*cpsG(manB)* R Infusion; pRH11
RH140	ACAGGAGGCCCCATATGGCAATCCACAATCGTGC	*pgm* F Infusion; pRH14
RH141	ACTCGAGATCTCATATGTTACGCGTTTTTCAGAA	*pgm* R Infusion; pRH14
